# IL-6, IL-10 and TNFα do not improve early detection of post-endoscopic retrograde cholangiopancreatography acute pancreatitis: a prospective cohort study

**DOI:** 10.1038/srep33492

**Published:** 2016-09-19

**Authors:** Mar Concepción-Martín, Cristina Gómez-Oliva, Ana Juanes, Josefina Mora, Silvia Vidal, Xavier Díez, Xavier Torras, Sergio Sainz, Candid Villanueva, Antoni Farré, Carlos Guarner-Argente, Carlos Guarner

**Affiliations:** 1 Gastroenterology Department. Hospital de la Santa Creu i Sant Pau, Institut de Reçerca-IIB Sant Pau, Universitat Autònoma de Barcelona, Barcelona, Spain; 2 Pharmacology Department. Hospital de la Santa Creu i Sant Pau, Institut de Reçerca-IIB Sant Pau, Universitat Autònoma de Barcelona, Barcelona, Spain; 3 Clinical Chemistry department. Hospital de la Santa Creu i Sant Pau, Institut de Reçerca-IIB Sant Pau, Universitat Autònoma de Barcelona, Barcelona, Spain; 4 Immunology Department. Hospital de la Santa Creu i Sant Pau, Institut de Reçerca-IIB Sant Pau, Universitat Autònoma de Barcelona, Barcelona, Spain; 5 Centro de Investigación Biomédica en Red en el Área temática de Enfermedades Hepáticas y Digestivas (CIBERhed) Instituto Carlos III, Madrid, Spain

## Abstract

The most reliable indicators for post-ERCP acute pancreatitis are elevated amylase levels and abdominal pain 24 hours after ERCP. As ERCP is often performed on an outpatient basis, earlier diagnosis is important. We aimed to identify early predictors of post-ERCP pancreatitis. We prospectively analyzed IL-6, IL-10, TNFα, CRP, amylase and lipase before and 4 hours after ERCP, and studied their association with abdominal pain. We included 510 patients. Post-ERCP pancreatitis occurred in 36 patients (7.1%). IL-6, IL-10, TNFα and CRP were not associated with post-ERCP pancreatitis. Levels of amylase and lipase were higher in patients with pancreatitis (522 U/L and 1808 U/L vs. 78 U/L and 61 U/L, respectively; p < 0.001). A cut-off of 218 U/L for amylase (x2.2 ULN) and 355 U/L for lipase (x6 ULN) had a negative predictive value of 99.2% and 99.5%, respectively. Amylase and lipase present a good correlation (Pearson coefficient 0.912). Among 342 (67.1%) patients without abdominal pain at 4 hours, post-ERCP pancreatitis was diagnosed in 8 (2.3%). Only 4 of these patients presented amylase or lipase > 3 ULN. Amylase and lipase were the only markers of post-ERCP pancreatitis 4 hours after the procedure.

Most cases of post-ERCP acute pancreatitis (PEP) are mild or moderate but up to 10% may be severe and potentially fatal^1^. Elevated amylase levels together with abdominal pain 24 hours after ERCP seem to be the most reliable indicators for this complication^2^,^3^. As many procedures today are performed on an outpatient basis, early diagnosis of PEP is vital to identify patients who should be hospitalized. The European Society of Gastrointestinal Endoscopy (ESGE) guidelines suggest serum amylase or lipase should be tested in patients who have pain 2–6 hours after ERCP and are to be discharged on the day of the procedure^1^. Patients with amylase values less than 1.5 times the upper limit of normality (ULN) or lipase less than 4 times this limit can be discharged without concern about risk of PEP. However, the observation of post-ERCP hyperamylasemia is common and often a benign phenomenon after ERCP in patients without PEP (25–75%)^4^,^5^,^6^,^7^, because there might be subclinical pancreatic damage. For this reason, we need other serological markers to improve early prediction of PEP.

Trypsinogen, trypsinogen activation peptide, C-reactive protein (CRP), and some cytokines have previously been evaluated to predict PEP. Cytokines might have special relevance because local production of inflammatory mediators has been observed in acute pancreatitis. Tumor necrosis factor α (TNFα), interleukin (IL)-1β, IL-6, and IL-8 are increased in this setting. Serum levels of anti-inflammatory molecules, such as IL-10, IL-1β receptor antagonist, and soluble IL-2 receptor (aIL-2r), are also significantly higher in acute pancreatitis. A dynamic balance between proinflammatory and anti-inflammatory cytokines has been observed^8^,^9^,^10^,^11^. The study aimed to evaluate early blood markers to predict PEP, and the association of these markers with abdominal pain.

## Methods

### Study design

A cohort of patients who underwent ERCP and were enrolled in a previously published clinical trial^12^ was used for this study. The current study is a secondary objective of that trial. All patients were 18 years or older. We excluded patients with previous sphincterotomy or chronic pancreatitis because of their low risk of PEP. Patients with ongoing acute pancreatitis were also excluded. Other exclusion criteria are detailed in the previous report^12^. The study protocol was approved at the Clinical Research Ethics Committee of the Hospital de la Santa Creu i Sant Pau, Barcelona. All methods were performed in accordance with the relevant guidelines and regulations. The procedure was performed on an outpatient basis or in hospitalized patients, in accordance to current endoscopy guidelines. All eligible patients received oral and written information about the study and gave their written consent prior to inclusion. Data on patient demographics, endoscopic procedure features, complications, and follow-up were prospectively collected in a standardized data form. All ERCP procedures were performed by expert endoscopists assisted by endoscopy fellows in training at a tertiary institution. All endoscopies were performed under propofol sedation guided by the endoscopist, and with standard air insufflation. The guidewire cannulation method was used in all cases. After the procedure, patients were closely monitored for a minimum of 6 hours. Blood samples were collected before and 4 hours after the procedure. Analyses included IL-6, IL-10, TNFα, CRP, amylase and lipase.

### Biochemical and immunological analysis

Blood samples were collected from all patients according to the protocol and serum was separated by centrifugation at 3500 rpm for 20 min. Serum amylase, lipase and CRP concentrations were determined on the day of sampling, and serum cytokines were determined on serum samples that were stored at −70 °C until analyzed. Serum amylase, lipase and CRP were measured on the automated Architect® analyser (Abbott Laboratories, Wiesbaden, Germany) using spectrophotometric assays for amylase and lipase and the immunoturbidimetric assay for CRP. Amylase values < 100 U/L, lipase < 60 U/L and CRP < 5 mg/L were considered normal.

Serum IL-6, IL-10 and TNF-α were measured using enzyme-chemiluminometric assays on the automated Immulite® 1000 analyser (Siemens Healthcare Diagnostics Products Ltd. Llanberis, UK). Reference ranges were ≤4 ng/L for IL-6, ≤9 ng/L for IL-10 and ≤8 ng/L for TNF-α in accordance with manufacturer’s data based on healthy controls. Analytical sensitivity was 2 ng/L for IL-6, 1 ng/L for IL-10, and 1.7 ng/L for TNF-α, and calibration range was up to 1000 ng/L for the three assays. Mean intra- and inter-assay coefficients of variations for each assay were 5.1% and 7.5% for IL-6, 4.2% and 9.9% for IL-10, and 2.6% and 6.5% for TNF-α, respectively.

### Follow up

All patients were evaluated by the gastroenterology physicians 4 hours after the procedure and outpatients were discharged at 6 hours if no signs or symptoms of complications were observed. In accordance with routine practice, patients with a suspected complication were admitted to the gastroenterology department. In these cases, blood testing and/or computed tomography (CT) scans were performed according to the hospital protocols. Additional samples were collected 24 hours after the procedure only if PEP was suspected. Seven days after the procedure, all patients were contacted by telephone to evaluate their post-procedure course. Participants with confirmed complications were followed until resolution.

### Definitions

PEP was defined according to the criteria established by Cotton *et al*.: abdominal pain with amylase level at least three times the upper limit of normality 24 hours after the ERCP, and requiring admission or prolongation of planned admission to at least 2 days^2^. The severity of pancreatitis was graded as mild when hospitalization was required for 2–3 days, moderate when it was required for 4 to 10 days and severe when it was prolonged more than 10 days, when it had necro-haemorragic features or pseudocysts, or when it required endoscopic, percutaneous or surgical intervention. Asymptomatic hyperamylasemia was defined as an increase in serum amylase concentrations by at least threefold the upper limit of normality, without symptoms of pancreatitis.

### Statistical analysis

Continuous variables were described as mean ± standard deviation when normally distributed and as median (interquartile range; IQR) when skewed. Proportions were used for categorical variables. Correlation between continuous variables was analyzed with the Pearson correlation coefficient. The association between blood markers and PEP was analyzed using non-parametric tests. Markers with statistical and clinical significance were analyzed with ROC curves to estimate an appropriate cut-off. All statistical analyses were performed using the SPSS Statistical Package (version 22.0, SPSS Inc., Chicago, IL).

## Results

### General characteristics

From May 2009 to February 2013, 510 patients were analyzed. Table 1 shows participants’ demographic and baseline characteristics. The main indications for ERCP were choledocholithiasis (62.2%) and biliary malignant stricture (31%). Nearly half of the procedures were performed on an outpatient basis (45.5%).

Table 2 shows the endoscopy characteristics. Deep biliary cannulation rate was 93.5%. Cannulation was difficult in 17.5% of procedures (>15 attempts of cannulation or cannulation failure). All patients completed the follow-up. The most common complication, PEP occurred in 36 patients (7.1%).

### Post-ERCP pancreatitis blood predictors

**Figure 1:**
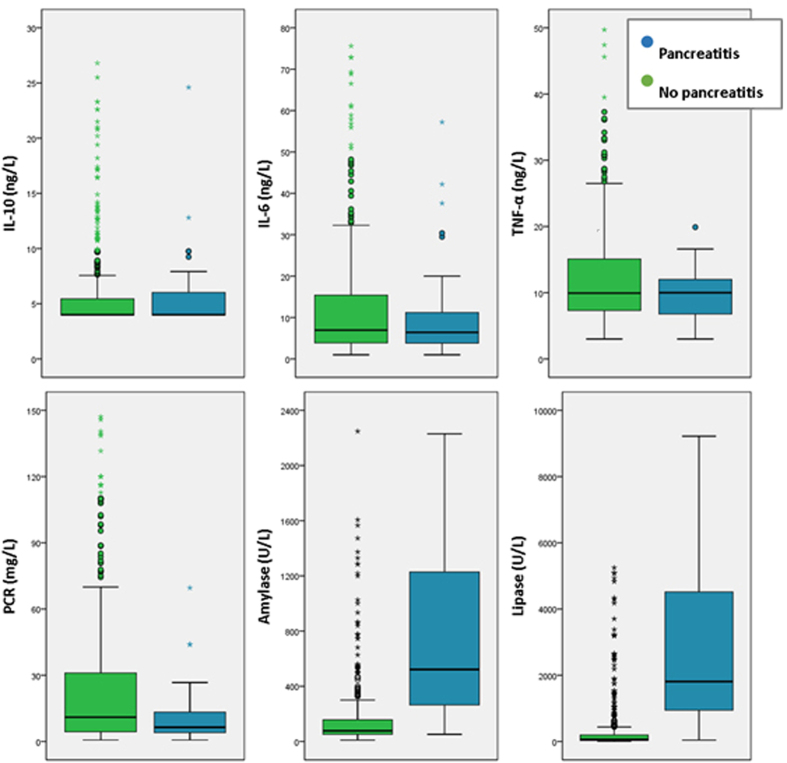
Potential early markers in serum levels 4 hours post-ERCP.

IL-6, IL-10 and TNFα were analyzed in 450 patients and CRP was analyzed in 480. IL-6, IL-10, TNFα and CRP levels 4 hours after ERCP did not differ in patients with PEP versus patients without PEP (Fig. 1). Neither did we observe differences between these two groups regarding incremental values from baseline of these markers. Evaluating only patients with normal pre-procedure values of IL-6, IL-10, TNFα and CRP we did not find any difference between patients with or without PEP. In patients with a previous inflammatory condition such as cholangitis or other infections, we observed that TNFα, CRP and leukocytes were elevated prior to ERCP, but remained stable 4 hours after the procedure. None of these patients developed PEP. There were no differences in other markers. We also analyzed the cohort with and without prophylaxis with somatostatin, and we did not find differences.

Amylase and lipase were analyzed 4 hours post-ERCP in 506 and 503 patients, respectively. Levels for both markers were significantly higher in patients with PEP. In these patients, amylase increased from basal levels of 78 U/L (52–159) to 522 U/L (263–1245). Lipase increased from basal levels of 61 U/L (30–197) to 1808 U/L (929–4692). Both markers presented statistically significant differences; p < 0.001 (Fig. 1). Amylase and lipase present a good correlation (Pearson coefficient 0.912).

Excluding patients with PEP, hyperamylasemia was observed in 57 patients (11.2%). Twenty-two of the 57 (38.6%) presented abdominal pain at 4 hours, attributed to air distension.

**Figure 2:**
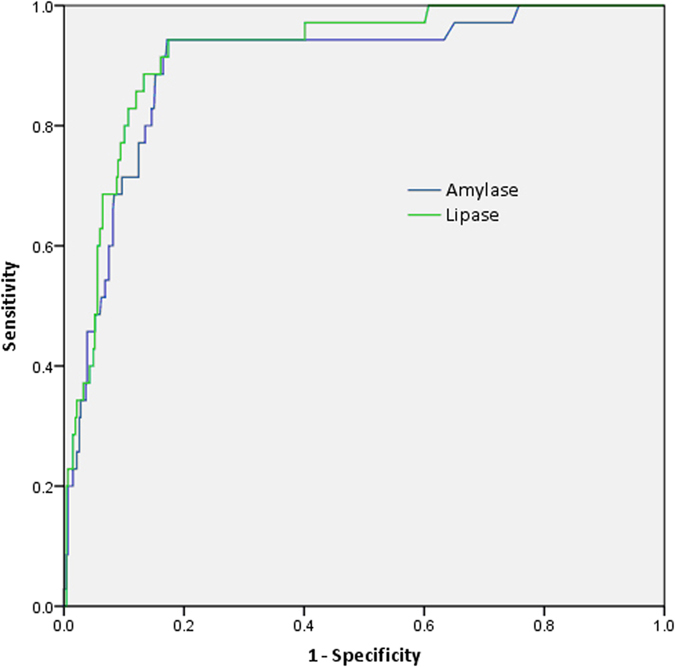
ROC curves to predict pancreatitis at 4 hours for amylase and lipase.

Figure 2 shows the ROC curves to predict pancreatitis according to amylase and lipase levels. ROC values were 0.89 and 0.92, respectively, showing good discriminative ability. A cut-off of 218 U/L for amylase (x2.2 times the upper limit of normality [ULN]) showed a sensitivity of 91.7%, a specificity of 82.5% and a negative predictive value (NPV) of 99.2%. A cut-off of 355 U/L for lipase (x6 ULN) showed a sensitivity of 94.4%, a specificity of 81.6%, and a NPV of 99.5% (Table 3).

### Blood predictors and Post-ERCP pancreatitis severity

The 21 patients with mild pancreatitis were compared with 15 with moderate or severe pancreatitis. IL-6, IL-10, TNFα and CRP levels 4 hours after ERCP did not differ among groups. The mean levels of lipase and amylase were slightly higher in the second group, without statistical differences.

### Abdominal pain and blood predictors

Abdominal pain 4 hours after the ERCP was observed in 168 patients (32.9%). In this group, 28 patients (16.7%) had PEP in contrast with 8 (2.3%) of the 342 (67.1%) patients without abdominal pain. Abdominal pain was therefore associated with PEP (p < 0.001); among the 36 patients with PEP, 28 patients (77.8%) had abdominal pain at 4 hours and 8 (22.2%) did not. Among the 8 cases of PEP without abdominal pain, amylase and lipase levels 4 hour after ERCP were normal in 2 patients, elevated only by 2–3 times ULN in another two, and elevated > 3 times ULN in 4.

ROC curves to predict pancreatitis at 4 hours for amylase and lipase in patients without abdominal pain had a value of 0.79 and 0.86 respectively, compared to 0.91 and 0.92 in patients with abdominal pain. Table 3 shows the sensitivity, specificity, positive and negative predictive values, and accuracy for the preferred cut-offs, and their association with abdominal pain. In patients with abdominal pain the preferred cut-off for amylase was 218 U/L (x2.2 ULN) and the preferred cut-off for lipase was 522 U/L (x8.7 ULN). Sensitivity and positive predictive values for amylase and lipase decreased in patients without pain.

## Discussion

We found that IL-6, IL-10, TNFα or CRP levels were not associated to post-ERCP acute pancreatitis (PEP) 4 hours after the procedure. In contrast, amylase and lipase were good early blood markers to predict PEP at 4 hours, especially when abdominal pain was present. In patients without abdominal pain, we observed a marked decrease in amylase and lipase sensitivity.

Several cytokines have been proposed to evaluate PEP. A study with 78 patients concluded that proinflammatory and anti-inflammatory cytokines increased significantly in the early stages of PEP, with IL-6 being the most useful^8^. IL-6 is produced by T-cells, macrophages, fibroblasts, and endothelial cells, and it is one of the main mediators in the inflammatory process. It induces the whole spectrum of acute phase proteins. Other cytokines play a more restricted role. Previous studies have assessed the potential of IL-6 to predict PEP^13^,^14^ and even its severity^14^,^15^ at 12–24 hours. Additionally, a study that included 30 patients with PEP found an increase in IL-6 levels in these patients at 6 and 24 hours^16^. However, in our series, IL-6 did not predict acute pancreatitis or its severity at 4 hours. Our result is consistent with two other studies that found differences at 8–12 hours or later, but not at 1 or 4 hours after the procedure^8^,^17^. Overall, although IL-6 might be a good predictor of PEP and its severity at 12 or 24 hours, it is not useful as a predictor at 4 hours.

IL-10, mainly secreted by T-cells, is a potent anti-inflammatory cytokine that inhibits several functions of macrophages and monocytes, including the production of IL-1, IL-6, IL-8, and TNF. It also decreases the cellular immune response by suppressing IL-2 and interferon-α production. Similarly to IL-6, it is significantly increased at 8 and 24 hours after ERCP^8^, and might predict acute pancreatitis severity more than 24 hours after the procedure^15^. However, this difference was not observed at 4 hours in our series.

TNFα is an inflammatory cytokine produced by T1 helper lymphocytes. Previous studies with this cytokine are contradictory, as some authors describe an increase in TNFα levels 8–12 hours after ERCP in patients with pancreatitis^8^,^13^, while others did not find significant differences^17^. Our results support these findings that TNFα is not a good early predictor for PEP.

CRP is an acute-phase protein of hepatic origin that increases following IL-6 secretion from macrophages and T cells. Probably for this reason, the peak of CRP is observed about 72 hours after the onset of acute pancreatitis^17^, considerably later than the previously commented cytokines. Therefore, although CRP determines the severity of post-ERCP pancreatitis at 12–24 hours and 36–48 hours^14^, this protein is a late marker.

In our study, amylase and lipase were the only early markers of pancreatitis 4 hours after ERCP especially in patients with abdominal pain. In a previous study, clinical assessment alone was unreliable in predicting PEP^18^. The authors reported that one third of patients who developed pancreatitis had no pain 2 hours after ERCP, whereas one third of patients who did not develop pancreatitis complained of pain. In contrast, amylase < 276 U/L (x2.4 ULN) and lipase < 1000 U/L (x4 ULN) were highly predictive in ruling out pancreatitis, with a NPV of 97 and 98%, respectively. Serum amylase values of 690 U/L (x6 ULN), or lipase of 17145 U/L (x70 ULN) predicted a probability of over 90% of developing pancreatitis^18^. In our study, amylase < 218 U/L (x2.2 ULN) and lipase < 522 U/L (x8.7 ULN) had a NPV of 99.1% in patients with abdominal pain.

Interestingly, previous studies suggested that lipase activity increased faster and higher than the activity of the other enzymes^5^,^7^,^17^,^19^. Overall, lipase seems to increase immediately after ERCP, even between 0 and 40 minutes, peaking at 6 hours after the procedure^17^,^19^. In one study with 70 patients, 8 of 9 patients with PEP presented lipase > 3x ULN at 0 h (immediately after ERCP). This value was significantly higher than in patients without pancreatitis^17^. Another previous retrospective study suggested that lipase was a more effective predictor than amylase at 4 hours^20^. In our study, ROC curves values for lipase were also slightly higher than for amylase. However, we observed that lipase and amylase levels had a good correlation 4 hours after ERCP, and additionally, lipase does not seem to be a useful marker regarding severity. A study by Kaw *et al*. showed no differences in lipase levels among severity groups at 12–24 and 36–48 hours^14^. Although lipase seems slightly superior to amylase at early stages, the two are comparable. Overall, the analysis of only amylase or lipase in this setting would be sufficient.

The number of patients with pancreatitis without abdominal pain at 4 hours was low (2.3%), and half of them presented normal or only slightly elevated levels of amylase and lipase. Therefore, PEP would have not been predicted in a routine blood test in these patients, possibly because it has a delayed onset in some patients. Fortunately, this occurs very rarely. In addition, 10.1% of our patients without abdominal pain and without pancreatitis presented hyperamylasemia. For these reasons, routine blood test 4 hours after ERCP in patients without abdominal pain might not be useful. Interestingly, one study observed increased levels of IL-6 and TNFα at 12 or 24 hours after ERCP in patients without PEP compared to basal levels^13^. These data support the existence of inflammatory activity and subclinical pancreatic damage in patients without PEP.

The major limitation of our study is that we only collected data 4 hours after ERCP. It could have been valuable for management of outpatients to collect samples at 6 and 8 hours after ERCP. Another limitation is that current prophylactic measures recommended in clinical guidelines (pancreatic stenting and endorectal indomethacin) were not used in this series. These measures might reduce the incidence of pancreatitis and modify the inflammatory markers. However, these measures were not clearly recommended when this study was designed. Moreover, a recent clinical trial did not report a benefit for endorectal indomethacin in 449 consecutive patients^21^. The main strengths of this study are the large sample and its prospective design. As previous studies about cytokines included fewer than 85 patients, the number of pancreatitis episodes (7 to 9) was often lower than in our study, limiting the interpretation of the results^8^,^13^,^17^. Moreover, in other studies, the exclusion of multiple patients may have biased results^14^,^16^.

In conclusion, amylase and lipase were the only early markers of PEP identified 4 hours after ERCP in our series. IL-6, IL-10, CRP and TNFα were not helpful in predicting this complication.

## Additional Information

**How to cite this article**: Concepción-Martín, M. *et al*. IL-6, IL-10 and TNFα do not improve early detection of post-endoscopic retrograde cholangiopancreatography acute pancreatitis: a prospective cohort study. *Sci. Rep.* **6**, 33492; doi: 10.1038/srep33492 (2016).

## Figures and Tables

**Table 1:** Demographic and baseline characteristics.

	Total(n** = **510)	PEP*(n = 36)	No PEP(n = 474)	*P* value
Age (years; mean ± SD)	73 ± 13	68 ± 15	73 ± 13	0.03
Gender				
Male	241 (47.3%)	15 (41.7%)	226 (47.7%)	0.49
Female	269 (52.7%)	21(58.3%)	248 (52.3%)	
Indication				
Choledocholithiasis	317 (62.2%)	22 (61.1%)	295 (62.2%)	0.59
Malignant stricture	158 (31%)	13 (36.1%)	145 (30.6%)	
Acute pancreatitis	20 (3.9%)	0	20 (4.2%)	
Sphincter Oddi dysfunction	4 (0.8%)	0	4 (0.8%)	
Others	11 (2.2%)	1 (2.8%)	10 (2.1)	
Previous acute pancreatitis	80 (15.7%)	8 (22.2%)	72 (15.2%)	0.26
Pancreas divisum	4 (0.8%)	0	4 (0.8%)	1
Hospitalization				
Inpatient	278 (54.5%)	19 (52.8%)	259 (54.6%)	0.83
Outpatient	232 (45.5%)	17 (47.2%)	215 (45.4%)	
Bilirrubin (μmol/L)	87 ± 129	84 ± 114	87 ± 130	0.86
Amylase (U/L)	64 ± 43	67 ± 29	63 ± 44	0.65
Lipase (U/L)	49 ± 61	64 ± 105	48 ± 56	0.37

^*^PEP: post-ERCP pancreatitis.

**Table 2:** ERCP characteristics and complications.

	Total(n = 510)	PEP*(n = 36)	No PEP(n = 474)	*P* value
Cholangiography	481 (94.3%)	36 (100%)	445 (93.9%)	0.25
Biliary duct diameter (mm; mean ± SD)	11 ± 4	11 ± 5	11 ± 4	0.45
Wirsung opacification	133 (26.1%)	12 (33.3%)	121 (25.5%)	0.3
Pancreatic acinarization	12 (2.4%)	2 (5.6%)	10 (2.1%)	0.21
Contrast injection (ml)	14 ± 8	13 ± 7	14 ± 8	0.92
>3 pancreatic duct injections	39 (7.6%)	4 (11.1%)	35 (7.4%)	0.34
Biliary sphincterotomy	465 (91.2%)	35 (97.2%)	430 (90.7%)	0.35
Biliary stent	152 (29.8%)	12 (33.3%)	140 (29.5%)	0.71
Precut sphincterotomy	78 (15.3%)	5 (13.9%)	73 (15.4%)	0.81
Cannulation difficulty				
1–5 attempts	310 (60.8%)	18 (50%)	292 (61.6%)	0.33
6–15 attempts	111 (21.8%)	11 (30.6%)	100 (21.1%)	
>15 attempts	89 (17.4%)	7 (19.4%)	82 (17.3%)	
Cannulation failure at first endoscopy	33 (6.5%)	1 (2.8%)	32 (6.8%)	0.5
Intradiverticular papilla	86 (16.9%)	3 (8.3%)	83 (17.5%)	0.25
Procedure time (minutes)	30 ± 21	31 ± 17	30 ± 21	0.94
Complications				
Acute pancreatitis	36 (7.1%)			
-Severe	2 (5.6%)			
-Moderate	13 (36.1%)			
-Mild	21 (58.3%)			
Bleeding	21 (4.1%)			
Cholangitis	12 (2.4%)			
Perforation	3 (0.6%)			
Deceased	1 (0.2%)			

^*^PEP: post-ERCP pancreatitis.

**Table 3:** Sensitivity, specificity, PPV, NPV, and accuracy for the preferred cut-offs to predict post-ERCP acute pancreatitis.

	Sensitivity	Specificity	PPV	NPV	Accuracy
All patients					
Amylase (218 U/L)	91.7%	82.5%	28.2%	99.2%	82.9%
Lipase (355 U/L)	94.4%	81.6%	28.1%	99.5%	82.5%
Abdominal pain					
Amylase (218 U/L)	96.4%	75.7%	44.3%	99.1%	79.2%
Lipase (522 U/L)	96.4%	79.3%	48.2%	99.1%	82.1%
No abdominal pain					
Amylase (231 U/L)	75%	87.2%	12.5%	99.3%	86.9%
Lipase (355 U/L)	75%	85.6%	11.3%	99.3%	85.4%
